# The case for more prudent prescribing of fluoroquinolones in primary care

**DOI:** 10.1080/13814788.2025.2584496

**Published:** 2025-11-10

**Authors:** Kate Browne, Juan Garcia Burgos, Eva Jirsová, Robin Ruepp, Gernot Bonkat, Shlomo Vinker, Walter Marrocco, Tiago Villanueva

**Affiliations:** ^a^ European Medicines Agency, Stakeholders and Communication, Amsterdam, The Netherlands; ^b^ State Institute for Drug Control, Prague, The Czech Republic; ^c^ European Medicines Agency, Human Medicines Division, Amsterdam, The Netherlands; ^d^ European Association of Urology, Arnhem, The Netherlands; ^e^ Department of Urology and Pediatric Urology, RWTH Aachen University, Aachen, Germany; ^f^ WONCA Europe, Ljubljana, Slovenia; ^g^ European Forum for primary care, Utrecht, the Netherlands; ^h^ European Union of General Practitioners / Family Physicians, Brussels, Belgium

Dear Editor

We read with interest the qualitative study by Soudais et al. which explores general practitioners’ (GPs) experiences and behaviours in the diagnostic and therapeutic management of male urinary tract infections (mUTIs) in France [1]. The study offers valuable insights into the clinical uncertainty surrounding the management of male cystitis and highlights a tendency towards empirical and prolonged fluoroquinolone use for the treatment of mUTIs.

## Definition and classification

The clinical uncertainty is particularly relevant in light of recent, fundamental shifts in the classification of UTIs. The long-held dogma that any mUTI is inherently ‘complicated’ has been officially superseded. The 2025 European Association of Urology (EAU) Guidelines have moved away from the ‘uncomplicated/complicated’ dichotomy to a more clinically relevant distinction between ‘localised’ and ‘systemic’ UTIs, a framework that applies to all sexes [2,3]. Concurrently, the 2025 Infectious Diseases Society of America (IDSA) guidelines, while retaining the terminology, have redefined ‘uncomplicated UTI’ to include ‘infection confined to the bladder in afebrile women or men’ [4]. This transatlantic consensus reframes the management of afebrile male cystitis and fundamentally challenges the rationale for treating it as an inherently severe disease requiring an aggressive empirical approach.

## European guideline warns

The 2025 EAU guideline on urological infections notes that cystitis in men without prostatic involvement is uncommon. While it recommends treatment with agents possessing good prostatic tissue penetration, mentioning trimethoprim-sulfamethoxazole or fluoroquinolones as possibilities, it places a critical condition that their use must be justified by susceptibility testing [3].

This requirement for culture-based, targeted therapy is reinforced by clinical reviews emphasising that a pre-treatment urine culture is essential in men due to a more varied and unpredictable microbiology [5].

Importantly, the guideline recommendation to use trimethoprim-sulfamethoxazole or fluoroquinolones must be interpreted in the context of overarching safety warnings and stewardship principles. The same EAU guideline strongly cautions that for cystitis, fluoroquinolones should only be used when other commonly recommended agents are considered inappropriate, a position that aligns with the stringent restrictions implemented by the European Medicines Agency (EMA) due to the risk of disabling, prolonged and potentially irreversible adverse drug reactions (ADRs) [3]. Therefore, the guidelines’ message is not an endorsement of routine fluoroquinolone use but rather a call for a highly selective, culture-guided approach that prioritises antimicrobial stewardship over the empirical use of this critical antibiotic class.

The study by Soudais et al. reflects a pattern of empirical use of fluroquinolones that is not unique to mUTIs, but part of a wider prescribing behaviour that has prompted regulatory scrutiny and action [6].

## Serious adverse reactions

In 2018, following a comprehensive benefit-risk evaluation, EMA concluded that systemic and inhaled fluoroquinolones are associated with serious, prolonged and potentially irreversible ADRs, predominantly affecting the musculoskeletal and nervous system [7]. These very rare ADRs include tendonitis, tendon rupture, neuropsychiatric events and neuropathies associated with paraesthesia. Tendonitis and tendon rupture, particularly of the Achilles tendon, can occur within 48 h of initiating treatment or several months after discontinuation. As a result of EMA’s recommendations, the European Commission implemented EU-wide risk minimisation measures (RMMs), which included restrictions on therapeutic indications and updates to the product information with descriptions of these ADRs and precautions for use. A direct healthcare professional communication (DHPC) was also disseminated to inform healthcare professionals accordingly [7].

The findings of a drug utilisation study assessing the impact of the RMMs suggest that fluoroquinolones may still be prescribed outside the licenced indications recommended by EMA. However, the study was subject to methodological constraints that precluded a precise quantification of such off-label use [6]. Another DHPC was subsequently disseminated to a broad range of healthcare professionals in 2023 to remind them of the legally binding restrictions [8].

## Patient counselling

The risk of ADRs must be weighed against the benefits of treatment with fluoroquinolones, particularly in older patients, those with renal impairment, or individuals taking corticosteroids, who are at increased risk of tendon injury. Patients should be counselled to discontinue treatment at the first sign of musculoskeletal or neurological symptoms and to contact their prescriber immediately. Furthermore, fluoroquinolones should be avoided in patients with a history of serious ADRs with fluoroquinolone or quinolone antibiotics; treatment should only be initiated in this cohort in the absence of alternative treatment options and following a careful evaluation of the benefit–risk balance [7].

## Recommendations for general practice

While fluoroquinolones remain a valid option in specific clinical scenarios, their safety profile and regulatory restrictions call for careful, indication-specific prescribing. We suggest that GPs familiarise themselves with the new recommendations for use and updated product information, consult local and national guidelines for antibiotic use, engage with local antibiotic stewardship initiatives and clinical decision support systems, if available, and prescribe fluoroquinolone-sparing regimens only when clinically appropriate. Furthermore, we would like to emphasise the importance of reporting suspected ADRs associated with fluoroquinolones through national reporting systems to facilitate continuous safety monitoring of these antibiotics.
